# Supplementary material for: Altered metabolic landscape in IDH‐mutant gliomas affects phospholipid, energy, and oxidative stress pathways
Source: EMBO Mol Med. 2017 Oct 20;9(12):1681–95. doi: 10.15252/emmm.201707729 (PMC5709746; doi:10.15252/emmm.201707729)
Supplement: Supplementary file 1 — Expanded View Figures PDF [file EMMM-9-1681-s001.pdf]

Expanded View Figures

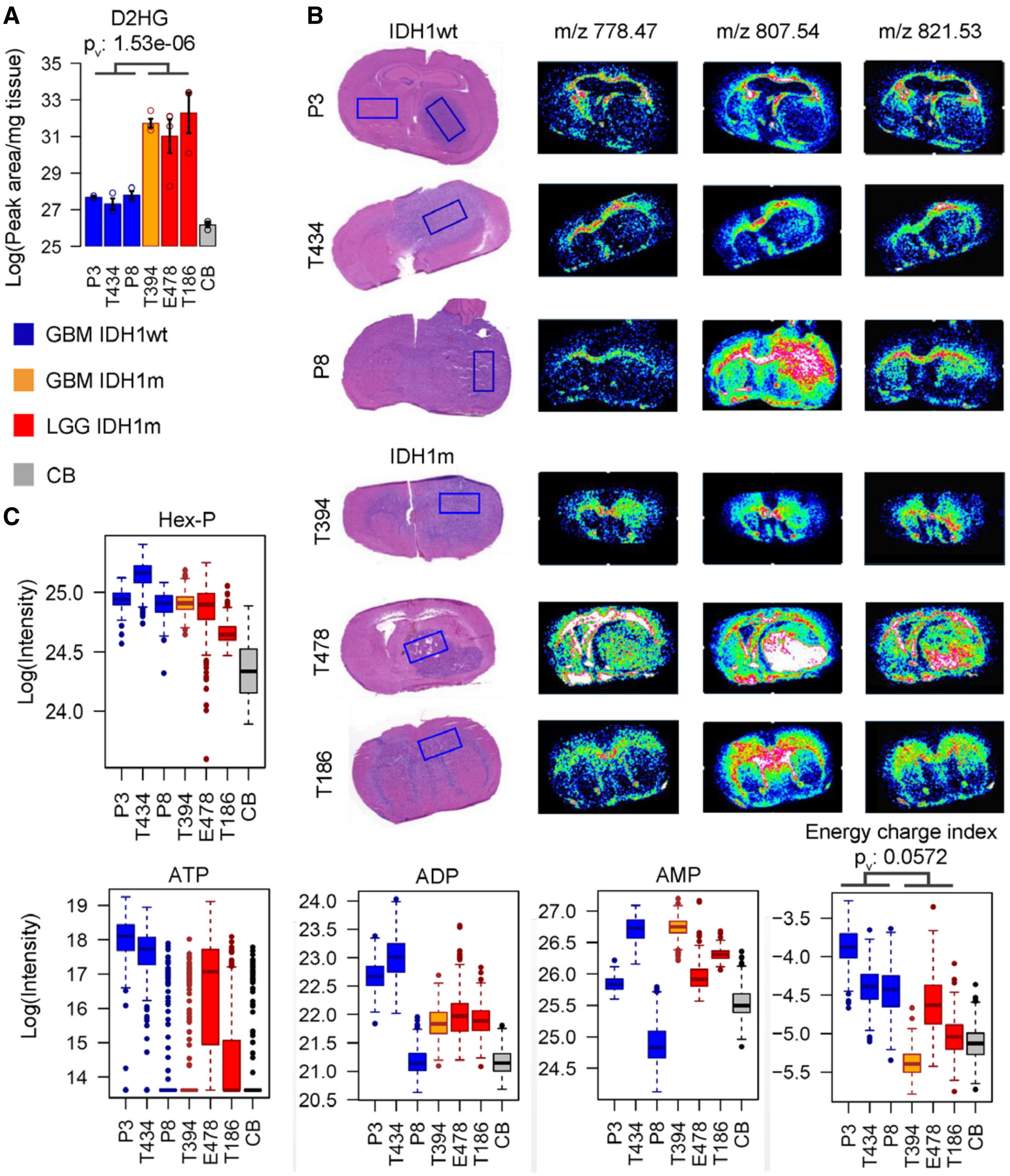

Figure EV1. Complementary metabolite quantifications.

A Quantification of D2HG metabolite distribution by LC-MS in six PDX with IDH1wt and IDH1m status (n = 3/sample/group) and in control brain (CB). Error bars on histograms represent standard error of the mean.

B Distributions of three phospholipidic compounds in different PDX as determined by MSI (as in Fig 1). Note that the histological sections are identical to the ones shown in Fig 1B.

C MALDI imaging quantification of indicated metabolites in six PDX and contralateral control brain (CB). Box limits indicate the 25th and 75th percentiles and center lines show the medians as determined by R software; whiskers represent the extreme low and high observed values, unless those are above 1.5 times interquartile range (IQR) – thereby whiskers are limited to 1.5 IQR. All outlying data points are represented by dots. Energetic charge defined as the ratio (ATP+1/2ADP)/(ATP+ADP+AMP) was calculated at each pixel. AMP, ADP, ATP: adenosine mono-, di-, and triphosphate, respectively, Hex-P: hexose phosphate.

Data information: *P*-values (p_v) are based on *t*-test.

 © 2017 The Authors

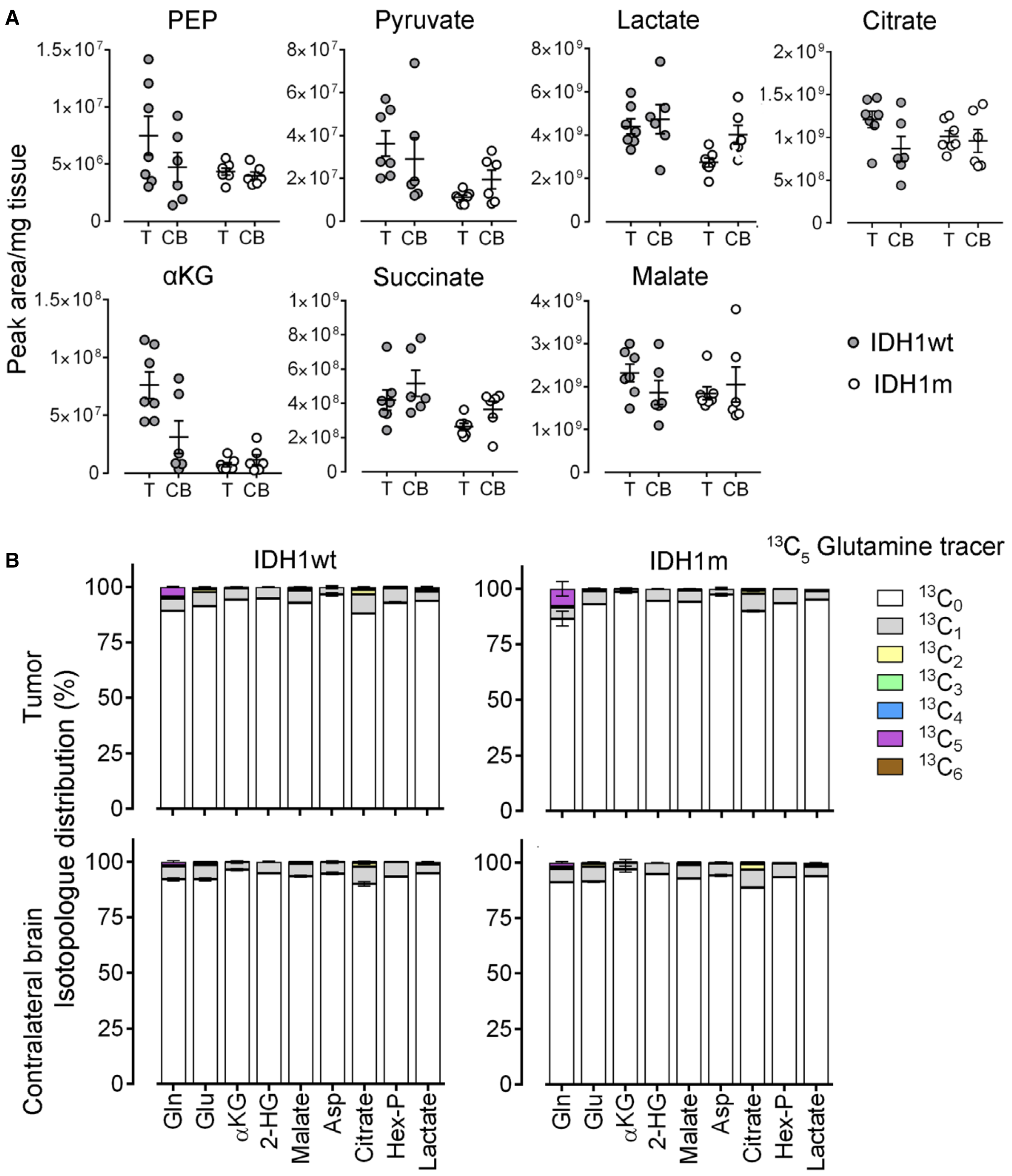

Figure EV2. Glucose and Glutamine tracer experiments.

A LC-MS quantification of steady-state levels of glycolysis and TCA cycle metabolites in tumor (T) and contralateral brain (CB) from $^{13}C_6$-glucose *in vivo* flux experiment shown in Fig 3C and D (n = 6/group). Error bars represent standard error of the mean.

B In vivo flux analysis after $^{13}C_5$-glutamine injection in PDX (n = 3/group). LC-MS quantification of the mass-isotope fractions after incorporation of ^{13}C (relative fractions of specific mass-isotopes compared to quantity of total metabolite). Error bars represent standard error of the mean.

© 2017 The Authors

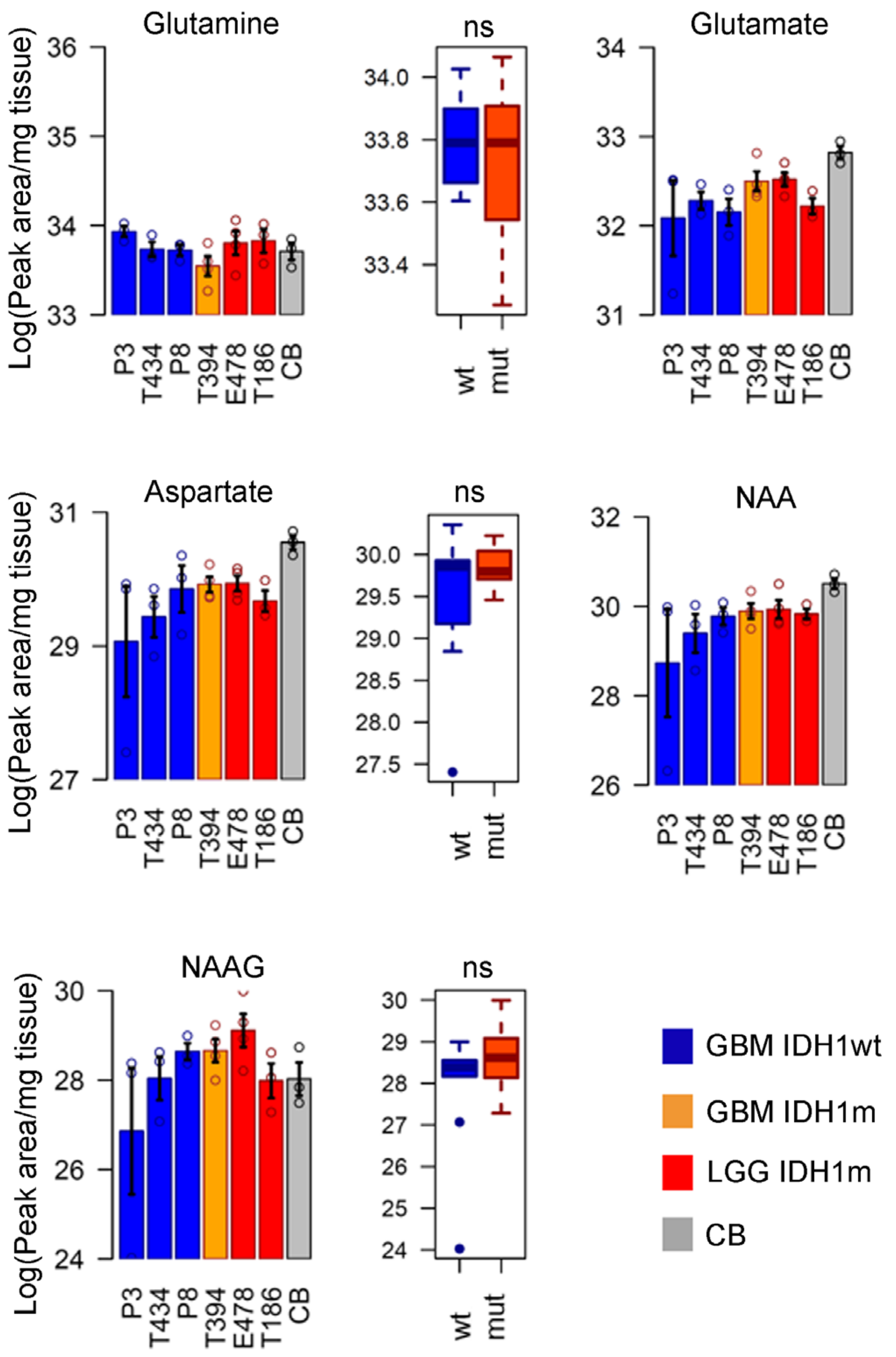

Figure EV3. Quantification of selected metabolites in tissue extracts.

Quantification of metabolite distribution from independent samples by LC-MS in six PDX with IDH1wt and IDH1m status and in normal brain (CB) (n = 3/samples/group). Error bars in histograms represent standard error of the mean. Metabolite levels in IDH1wt and IDH1m groups are compared by t-test. ns: non-significant. Box limits indicate the 25th and 75th percentiles and center lines show the medians as determined by R software; whiskers represent the extreme low and high observed values, unless those are above 1.5 times interquartile range (IQR) – thereby whiskers are limited to 1.5 IQR. All outlying data points are represented by dots. NAA: N-acetylaspartic acid; NAAG: N-acetylaspartylglutamic acid.

 © 2017 The Authors

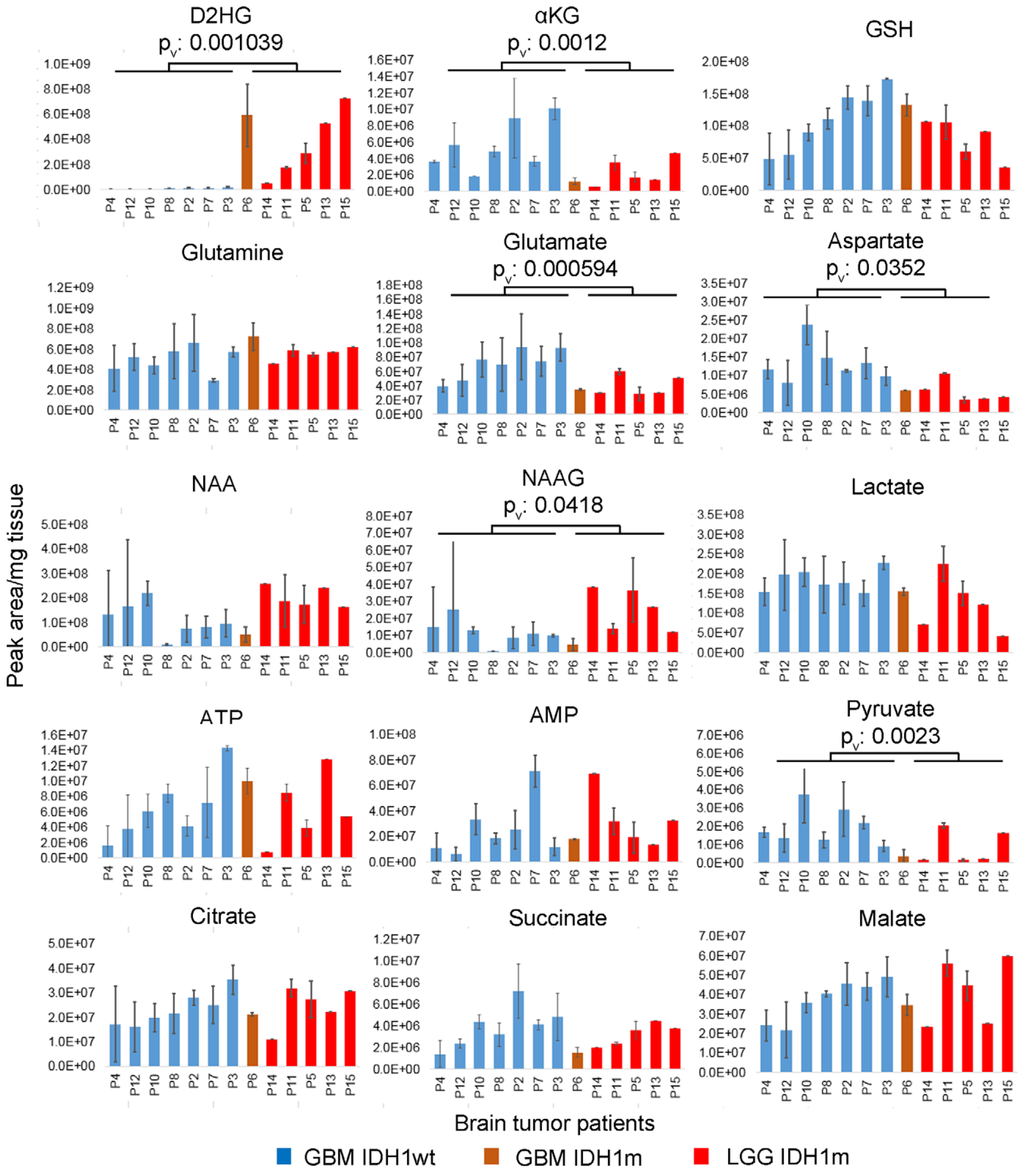

Figure EV4. Metabolites in clinical glioma tissue extracts.

LC-MS analysis of selected metabolites in clinical tumor extracts from glioma patients (P2 to P15) with IDH1wt (n = 7) and IDH1m (n = 6) status. IDHwt GBM in blue, IDHm GBM in orange, IDHm LGG in red. Error bars represent standard error of the mean. Results of statistical comparison of IDH1wt and IDH1m groups are indicated, P_v: P-values. NAA: N-acetylaspartic acid; NAAG: N-acetylaspartylglutamic acid.
