# Supplementary material for: Altered metabolic landscape in IDH‐mutant gliomas affects phospholipid, energy, and oxidative stress pathways
Source: EMBO Mol Med. 2017 Oct 20;9(12):1681–95. doi: 10.15252/emmm.201707729 (PMC5709746; doi:10.15252/emmm.201707729)
Supplement: Supplementary file 2 — Table EV1 [file EMMM-9-1681-s002.docx]

| **Table ev1: pairwise comparison of samples based on untargeted MSI analysis.**  Statistical analysis was done on different regions of interest (ROI) (between 50 and 59 spectra/ROI) based on 300 main peaks of each spectrum. Data shown are from pairwise comparisons of five ROIs: two Tumour ROI (IDH1wt_T, IDH1m_T); two contralateral brain (IDH1wt_CB, IDH1m_CB) and normal brain (NB); m/z based identification of metabolites is based on two databases (Imabiotech, Metlin); tolerance for identification in negative mode in Metlin: 10ppm. T: tumour; CB: contralateral brain; NB: normal brain. | | | | |
| --- | --- | --- | --- | --- |
| **Pairwise Comparison** |  |  | **Metabolite Identities** | |
| **Condition 1 vs Condition 2** | **Number of differential m/z** | **Number of m/z with increased intensity** | **IMABIOTECH** | **METLIN** |
| **IDH1wt vs IDH1m_T** | 95 |  | 10 | 217 |
| IDH1w_T |  | 48 | 6 | 65 |
| IDH1m_T |  | 47 | 4 | 152 |
| **IDH1wt_T vs NB** | 79 |  | 4 | 411 |
| NB |  | 42 | 1 | 120 |
| IDH1wt_T |  | 37 | 3 | 291 |
| **IDH1wt vs CB** | 78 |  | 3 | 257 |
| IDH1wt_CB |  | 19 | 1 | 129 |
| IDH1wt_T |  | 59 | 2 | 128 |
| **IDH1m vs NB** | 119 |  | 11 | 439 |
| NB |  | 53 | 4 | 294 |
| IDH1m_T |  | 66 | 7 | 145 |
| **IDH1m Tumor vs CB** | 86 |  | 8 | 300 |
| IDH1m_CB |  | 34 | 5 | 174 |
| IDH1m_T |  | 52 | 3 | 126 |
