# Supplementary material for: Altered metabolic landscape in IDH‐mutant gliomas affects phospholipid, energy, and oxidative stress pathways
Source: EMBO Mol Med. 2017 Oct 20;9(12):1681–95. doi: 10.15252/emmm.201707729 (PMC5709746; doi:10.15252/emmm.201707729)
Supplement: Supplementary file 3 — Table EV2 [file EMMM-9-1681-s003.docx]

**Table EV2: Compounds with increased intensity in IDH1 mutant versus IDH1 wild type Tumor**

Differential candidate compounds as determined by untargeted MSI analysis, based on pair wise comparison shown in Table EV1 (present in tumor and normal brain). FC: Fold change difference in intensity; IDH1m_T = IDH1 mutant tumour; IDH1wt_T = IDH1 wild type tumor.

| **m/z** | **IDHm_T / IDH1wt_T  (FC)** | **ID in Metlin** |
| --- | --- | --- |
|  |  |  |
| 778.51 | 1421.62 | PE(16:0/22:6(54Z,7Z,10Z,12E,16Z,19Z)(14OH)) or C16 Sulfatide |
| 285.07 | 162.37 | Thr-Met |
| 776.49 | 239.36 | PE(20:3(8Z,11Z,14Z)/16:0) |
| 303.08 | 145.65 | NAAG |
| 805.52 | 118.02 | PI(P-18:0/15:1(9Z)) or PI(P-16:0/17:1(9Z)) or PI(O-16:0/17:2(9Z,12Z)) |
| 820.51 | 109.86 | PS(22:6(4Z,7Z,10Z,13Z,16Z,19Z)/17:0 to PS(17:2(9Z,12Z)/22:4(7Z,10Z,13Z,16Z)) |
| 804.52 | 51.47 | Scytophycin C or PC or PE (M+Cl) |
| 792.48 | 38.14 | PS(20:5(5Z,8Z,11Z,14Z,17Z)/17:1(9Z)) or PS(P-18:0/17:2(9Z,12Z)) |
| 779.51 | 35.21 | PI(O-16:0/15:1(9Z)) or PG(P-18:0/17:2(9Z,12Z)) |
| 795.5 | 33.09 | PI(18:0/13:0): M-H :>6 or PA |
| 794.5 | 17.56 | PS(19:1(9Z)/18:4(6Z,9Z,12Z,15Z)) M-H:>6 or C16(OH) Sulfatide |
| 823.53 | 12.47 | PI(19:0/14:0) or PG(18:3(9Z,12Z,15Z)/22:2(13Z,16Z)) |
| 245.05 | 8.90 | Phosphatidyl glycerol |
| 414 | 7.58 | 6-Thioguanosine monophosphate |
| 807.54 | 6.40 | PI(O-16:0/17:1(9Z)) |
| 822.53 | 6.21 | PS(17:1(9Z)/22:4(7Z,10Z,13Z,16Z)) |
| 719.53 | 5.50 | PG(O-16:0/17:1(9Z)) |
| 597.3 | 5.30 | PI(18:1(9Z)/0:0) |
| 821.52 | 5.21 | PI(13:0/20:1(11Z)) |
| 433.23 | 4.98 | PA(18:2(9Z,12Z)/0:0) |
| 647.46 | 4.21 | PA(16:0/16:0) |
| 806.54 | 4.16 | PE(18:0/22:6(4Z,7Z,10Z,12E,16Z,19Z)(14OH)) |
| 303.05 | 4.11 | Inosine |
| 436.25 | 4.06 | PC(O-10:1(9E)/2:0) or PE(15:1(9Z)/0:0) |
| 376.04 | 4.02 | Glucosisymbrin |
| 442.01 | 3.65 | Guanosine 5′-diphosphate (GDP) |
| 355.06 | 3.50 | protonated glutathione sulfonate GSO3- (?) |
| 322.04 | 3.48 | Cytidine 2'-phosphate |
| 362.05 | 3.42 | Caffeoyl C1-glucuronide |
| 683.33 | 3.41 | L-Alanyl-L-isoleucyl-L-leucyl-L-phenylalanyl-L-tryptophan |
