# Supplementary material for: Altered metabolic landscape in IDH‐mutant gliomas affects phospholipid, energy, and oxidative stress pathways
Source: EMBO Mol Med. 2017 Oct 20;9(12):1681–95. doi: 10.15252/emmm.201707729 (PMC5709746; doi:10.15252/emmm.201707729)
Supplement: Supplementary file 4 — Table EV3 [file EMMM-9-1681-s004.docx]

**Table EV3: Compounds with increased intensity in IDH1 wild type versus IDH1 mutant Tumor**

Differential candidate compounds as determined by untargeted MSI analysis, based on pair wise comparison shown in Table EV1 (present in tumor and normal brain). FC: Fold change difference in intensity; IDH1m_T = IDH1 mutant tumour; IDH1wt_T = IDH1 wild type tumor; ID: identification

| **m/z** | **IDH1wt_T / IDH1m_T  (FC)** | **ID in Metlin** |
| --- | --- | --- |
|  |  |  |
| 136.01 | 262.20 | no metabo |
| 199.94 | 91.98 | no metabo |
| 241.93 | 46.18 | no metabo |
| 891.63 | 43.35 | PI(P-18:0/21:0) or PI(P-20:0/19:0) or PI(O-20:0/19:1(9Z)) |
| 124.01 | 28.92 | Taurine |
| 890.63 | 17.14 | C24 Sulfatide |
| 150.02 | 15.02 | 6-Imino-5-oxocyclohexa-1,3-dienecarboxylate |
| 221.06 | 14.45 | Cys Thr or Cystathionine |
| 220.03 | 12.67 | N-Acetylcarbocysteine |
| 181.97 | 11.97 | Selenohomocysteine or Selenomethyl selenocysteine |
| 197.94 | 11.68 | 2-thiothiazolidine-4-carboxylic acid |
| 789.54 | 11.28 | PA(21:0/22:6(4Z,7Z,10Z,13Z,16Z,19Z)) |
| 128.89 | 10.08 | Thiadiphosphirene (?) |
| 194.01 | 8.39 | Carbocysteine sulfoxide |
| 889.61 | 8.06 | PI(P-20:0/19:1(9Z)) |
| 862.6 | 7.61 | C22 Sulfatide |
| 888.61 | 7.58 | C24:1 Sulfatide |
| 907.63 | 7.15 | PI(17:0/22:0) |
| 207.04 | 7.05 | Serinyl-Cysteine |
| 166.02 | 6.97 | Homocysteinesulfinic acid |
| 134.05 | 6.17 | Adenine |
| 152 | 6.15 | 3-Sulfinoalanine (CSA) |
| 788.53 | 5.64 | PE(18:1(11Z)/22:6(4Z,7Z,10Z,13Z,16Z,19Z)) or PC(22:6(4Z,7Z,10Z,13Z,16Z,19Z)/15:1(9Z)) |
| 178.02 | 5.48 | S-Carboxymethyl-L-cysteine |
| 906.62 | 5.08 | 3-O-Sulfogalactosylceramide or C24-OH Sulfatide |
| 186.04 | 4.93 | 2-(Acetamidomethylene)succinate |
| 140.01 | 4.10 | O-Phosphorylethanolamine |
| 737.48 | 4.06 | PA(21:0/15:1(9Z)) |
| 187.04 | 3.91 | 1-Hydroxy-2-naphthoic acid |
| 176.94 | 3.14 | Pyrophosphate or 2-Mercaptoethanesulfonic acid |
