# Supplementary material for: Altered metabolic landscape in IDH‐mutant gliomas affects phospholipid, energy, and oxidative stress pathways
Source: EMBO Mol Med. 2017 Oct 20;9(12):1681–95. doi: 10.15252/emmm.201707729 (PMC5709746; doi:10.15252/emmm.201707729)
Supplement: Supplementary file 5 — Table EV4 [file EMMM-9-1681-s005.docx]

**Table EV4: Metabolite characterization by fragmentation**

Confirmation of metabolite identity by fragmentation in targeted MS approach in negative and positive mode on tissue sections. [M-H]-: compound (m/z) detected in negative mode, [M+H]+: compound (m/z) detected in positive mode.

| **Nr** | **Name** | **Validation** | **[M-H]-** | **[M+H]+** | **Fragments negative mode** | **Fragments positive mode** |
| --- | --- | --- | --- | --- | --- | --- |
| 1 | aspartic acid | Sensitivity too low | 132.029134 | 134.044784 |  |  |
| 2 | acetylaspartylglutamate (NAAG) |  | 303.082292 | 305.097942 | 226.03/  182.04/  128.03/  96.01 |  |
| 3 | L-cystathionine |  | 221.059054 | 223.074704 | 134.03/  120.01 |  |
| 4 | Glutamine |  | 145.060769 | 147.076419 | 128.03 | 130.05/  84.04 |
| 5 | L-Glutamic acid |  | 146.044784 | 148.060434 | 128.03 |  |
| 6 | GSH |  | 306.075433 | 308.091083 | 288.06/  272.08/  254.08/  179.04/  128.03 |  |
| 7 | ascorbic acid |  | 175.023714 | 177.039364 | 115.00/  87.01 |  |
| 8 | N-acetyl-L-aspartic acid (NAA) |  | 174.039699 | 176.055349 | 130.05/  114.02/  88.04 |  |
| 9 | N-Acetylcysteine (NAC) | n.d. | 162.021941 | 164.037591 |  |  |
| 10 | Cysteine | n.d. | 120.011376 | 122.027026 |  |  |
| 11 | sn-glycero-3-Phosphoethanolamine | Ref | 214.0475 | 216.06315 |  |  |
| 12 | stearic acid |  | 283.263157 | 285.278807 |  |  |
| 13 | citric acid | or isocitric acid | 191.018629 | 193.034279 | 111.01 |  |
| 14 | N-palmitoyl taurine | n.d. | 362.235956 | 364.251606 |  |  |
| 15 | Cytidine |  | 242.077147 | 244.092797 |  | 112.05 |
| 16 | Uridine monophosphate (UMP) | Sensitivity too low | 323.027493 | 325.043143 |  |  |
