# Supplementary material for: Altered metabolic landscape in IDH‐mutant gliomas affects phospholipid, energy, and oxidative stress pathways
Source: EMBO Mol Med. 2017 Oct 20;9(12):1681–95. doi: 10.15252/emmm.201707729 (PMC5709746; doi:10.15252/emmm.201707729)
Supplement: Supplementary file 6 — Table EV5 [file EMMM-9-1681-s006.docx]

**Table EV5: Tissue exctinction coefficients (TEC) of selected metabolites using stable isotope labeled references**

Isotopically labeled reference compounds added to MALDI matrix to determine detection sensitivity on tissue sections and tissue extinction coefficient (TEC) values in different regions of interest (ROI). d3, d4: deuterated compound; ^13^C or ^15^N-labeled forms of compounds of interest. CL: contralateral hemisphere; NB: normal brain; T: tumour.

| ROI | NAA-d3 | L-cystathionine-d4 | NAAG-d3 | GSH 13C 15N | GSSG 13C 15N |
| --- | --- | --- | --- | --- | --- |
| IDHm CL | 0.33 | 0.27 | 0.21 | 0.17 | 0.09 |
| IDHwt CL | 0.35 | 0.32 | 0.24 | 0.20 | 0.12 |
| NB | 0.35 | 0.33 | 0.18 | 0.15 | 0.06 |
| IDHm T | 0.28 | 0.25 | 0.20 | 0.17 | 0.09 |
| IDHwt T | 0.29 | 0.32 | 0.23 | 0.22 | 0.18 |
