# Supplementary material for: Altered metabolic landscape in IDH‐mutant gliomas affects phospholipid, energy, and oxidative stress pathways
Source: EMBO Mol Med. 2017 Oct 20;9(12):1681–95. doi: 10.15252/emmm.201707729 (PMC5709746; doi:10.15252/emmm.201707729)
Supplement: Supplementary file 7 — Table EV6 [file EMMM-9-1681-s007.docx]

**Table EV6: Characterization of Clinical samples**

Patient diagnosis (clinical samples used in Figure EV4), histology, grade, IDH status (x: IDH1 mutation), 1p19q codeletion status and DNA methylation status (hypermethylated = GCIMP phenotype). GBM: glioblastoma. Note that P6 corresponds to an IDH mutant GBM.

| **Patient** | **Tumour type** | **Grade** | **IDH1 R132H** | **1p /19q codeletion** | **Hyper-methylated** |
| --- | --- | --- | --- | --- | --- |
| P2 | GBM | G4 |  |  |  |
| P3 | GBM | G4 |  |  |  |
| P4 | GBM | G4 |  |  |  |
| P5 | Oligodendroglioma | G2 | x | x | x |
| P6 | GBM | G4 | x |  |  |
| P7 | GBM | G4 |  |  |  |
| P8 | GBM | G4 |  |  |  |
| P10 | GBM | G4 |  |  |  |
| P11 | Astrocytoma | G2 | x |  |  |
| P12 | GBM | G4 |  |  |  |
| P13 | Oligodendroglioma | G2/G3 | x | x | x |
| P14 | Astrocytoma | G2 | x |  |  |
| P15 | Oligodendroglioma | G2 | x | x | x |
